# Coronavirus Disease 2019 Pandemic Effects on Overseas Pakistanis Particularly Residing in China, Saudi Arabia and United Kingdom

**DOI:** 10.3389/fpubh.2022.768812

**Published:** 2022-03-29

**Authors:** Tauseef Ahmad, Bibi Nazia Murtaza, Faizan Ahmad, Manal Abdulaziz Murad, Mukhtiar Baig, Arouba Imtiaz, Fizzah Baig, Javaria Baig, Muhammad Siraj, Abdullah Khalid Sagga

**Affiliations:** ^1^Department of Epidemiology and Health Statistics, School of Public Health, Southeast University, Nanjing, China; ^2^Department of Zoology, Abbottabad University of Science and Technology, Abbottabad, Pakistan; ^3^Department of Microbiology, Abbottabad University of Science and Technology, Abbottabad, Pakistan; ^4^Department of Family Medicine, Faculty of Medicine, King Abdulaziz University, Jeddah, Saudi Arabia; ^5^Department of Clinical Biochemistry, Faculty of Medicine Rabigh, King Abdulaziz University, Jeddah, Saudi Arabia; ^6^Cardiff Medical School, Cardiff, United Kingdom; ^7^Ziauddin Medical College-Ziauddin University, Karachi, Pakistan; ^8^Liaquat College of Medicine and Dentistry, Karachi, Pakistan; ^9^General Dentist Assistant Agency for Primary Health Care, Medical Program for Chronic Disease General Department, Ministry of Health, Riyadh, Saudi Arabia

**Keywords:** COVID-19, pandemic effects, overseas Pakistani's, psychological issues, cross-sectional study

## Abstract

**Aim:**

This study explored the Coronavirus Disease 2019 (COVID-19) pandemic effects on overseas Pakistani's residing in various parts of the world, particularly in China, Saudi Arabia (SA), and the United Kingdom (UK).

**Methods:**

This cross-sectional study was completed between November 2020 and April 2021. An online questionnaire was designed and circulated *via* various social media mediums to overseas Pakistani communities. The obtained data were statistically analyzed through SPSS version 19 for windows. *P* < 0.05 was considered statistically significant.

**Results:**

A total of 542 overseas Pakistani participated in the current study. In total, 157 (29%) were females and 385 (71%) males. There were 174 (32.1%), 142 (26.45%), 117 (21.6%), and 109 (19.85%) participants from the UK, SA, China, and other countries respectively. Some participants, or their family members, 93 (17.2%), got infected with the COVID-19. About one-third, 165 (30.4%), of the respondents were afraid that their company would violate their contracts or lose their jobs or be paid less. The majority, 469 (86.5%), believed that the lockdown is increasing their psychological stress. More than half of the participants, 314 (57.9%), stated that the Pakistani embassy did not facilitate them in their country of stay. About one-third, 194 (35.8%), of the respondents faced visa-related issues. More than one-third of respondents, 221 (40.8%), faced health issues due to lack of physical activities during the lockdown. Males were afraid that their company would violate their contract, lose jobs, or be paid less than females (*p* < 0.001). Both genders had psychological stress and health issues because of the pandemic. The participants from SA faced more visa-related issues, and they were less satisfied with the efforts of the Pakistani embassy to facilitate them compared to the UK and China participants (*p* = 0.013).

**Conclusion:**

Our data indicate that the COVID-19 pandemic impacted Pakistanis living in the UK, SA, China, and other parts of the world. They had health-related issues, visa problems and dissatisfaction with Pakistani embassy facilitations. Pakistanis living abroad require government assistance to resolve their issues.

## Introduction

In late December 2019, a highly transmissible and pathogenic coronavirus named Severe Acute Respiratory Syndrome Coronavirus 2 (SARS-CoV-2) emerged in Wuhan, China, and caused an outbreak of unusual viral pneumonia. The virus has expanded beyond the borders and caused a pandemic designated Coronavirus Disease 2019 (COVID-19). This ongoing COVID-19 pandemic has threatened health care systems, public safety, animal, environmental and human health (1–3). Because of the devastating effects of the COVID-19, overseas workers and their dependents are socially and economically vulnerable. Furthermore, lockdown, quarantine, self-isolation, and social distancing have significantly affected the overall mental, physical and social wellbeing (4, 5).

Pakistan has a population of ~2,200 million people, and ~8.4 million people live in other countries as immigrants or expatriates. Aside from that, thousands of Pakistanis are holding the student visas. Pakistan is one of the top 10 recipients of international remittances. In 2019, Pakistan received USD 21.8 billion in foreign remittances, accounting for ~8% of its GDP. In this way, overseas workers are directly contributing to the country's socio-economic development. The top five countries from where the overseas Pakistanis send remittances are Saudi Arabia (SA) (23%), United Arab Emirates (UAE) (21%), United Kingdom (UK) (16%), United States of America (USA) (16%), and other Gulf Cooperation Council (GCC) countries (10%) (6).

According to government sources, nearly 4.7 million Pakistanis are working in the Middle East (7). Many expatriates faced unprecedented challenges and psychological symptoms across geographies during the COVID-19 pandemic (8, 9). The migrant workers in several countries were suffering from unemployment, social discrimination, financial hardship, and mental stress during the COVID-19 pandemic (10). This demonstrates how the pandemic has significantly impacted people's mental health, particularly among expatriates. In the COVID-19 pandemic all over the world, expatriates and workers faced several problems.

However, the current COVID-19 pandemic effects are widespread and have forced enormous pressure on people from all backgrounds or professions, including healthcare workers, students, security forces, and the general public, thus resulting in high psychological symptoms including anxiety, depression, fear for their safety, higher levels of stress, insomnia, negativity, poor sleep and somatization (4, 11–14). Thus, the current study was conducted to explore the COVID-19 pandemic effects on overseas Pakistani's residing in various parts of the world, particularly in China, SA, and UK.

## Methodology

This cross-sectional study was conducted from November 2020 to April 2021, and data were collected from the overseas Pakistani people residing mainly in China, SA, UK, UAE, Malaysia, USA, Canada, and other countries.

A brief description was provided regarding the study questionnaire, its purpose, and instructions on how to complete it before completing the survey. Before proceeding further, all participants were required to express their willingness to volunteer for participation by answering yes or no and filling out the questionnaire was also considered their consent. Participants were asked if they would be willing to participate by answering “yes” or “no.”

Furthermore, if participants did not wish to continue, they were given the option to withdraw at any time. All participants were assured that their identity would not be disclosed, and data will be used only for research purpose. The sample size was calculated on sample size calculator from calculator.net (https://www.calculator.net/). There was a need of 385 samples to have a confidence level of 95% that the real value is within ±5% of the measured/surveyed value. The sample size was increased to improve validity and generalizability.

A snowball sampling technique was employed to recruit participants. Using Google Forms, an online self-reported questionnaire was created. The survey link was sent to the investigators' contacts *via* WhatsApp, Facebook, Instagram, WeChat, email, and other social media platforms, and participants were encouraged to forward it to Pakistani friends' circles residing in their country of stay.

The questionnaire had several parts. The first part contains demographic information about gender, education, employment status, country of stay and infection of coronavirus. There were several questions about the impact of the COVID-19 pandemic and lockdown like fear, anxiety, stress, fear of unemployment, and salary reduction. There were questions regarding their or their family members' infected with COVID-19 and treatment in a hospital in their country of stay. There were questions about the visa problems and facilitation by the Pakistani embassy in their country of stay. The consequences of lockdown on their health, their family health reside in Pakistan, and their satisfaction with the Pakistan government to manage pandemic in Pakistan. The format of the questionnaire was “yes,” “no,” or “maybe.”

The questionnaire was prepared in English and Urdu languages, and before distribution, its content validity was done by two senior professors and one medical educationist. It was modified as per their suggestions. A small pilot study was conducted, and participants were requested to point out any flaws and ambiguity in the questions. There were 40 participants in the pilot study. In this way, the comprehension and correctness of the questions were checked. The reliability of the questionnaire was calculated by Cronbach's alpha (Alpha = 0.78).

## Statistical Analysis

The information was gathered for each participant in Microsoft Excel 2019. The obtained data were analyzed using SPSS version 19 software for windows. The Chi-square test was applied to assess relationships between various variables. Moreover, the qualitative variables are expressed as frequencies and percentages. *P* < 0.05 was considered statistically significant.

## Results

A total of 542 overseas Pakistani, of which 157 (29%) were females and 385 (71%) males participated in the current study. In total, majority of the study participants are living in UK 174 (32.1%), followed by SA 142 (26.45%), China 117 (21.6%), USA 33 (6.15%), Canada 24 (4.4%), UAE 21 (3.9%), Malaysia 17 (3.1%), and other countries 13(2.4%). The socio-demographic characteristics and perceptions of study participants are presented in Table 1.

**Table 1:** Socio-demographic characteristics and perceptions of the study participants.

**Variables**		***N* (%)**
Gender	Female	157 (29)
	Male	385 (71)
Education	College	53 (9.8)
	Graduate	138 (25.5)
	Postgraduate	351 (63.8)
Employment status	Doctor	128 (23.6)
	Businessman	53 (9.8)
	Teacher	96 (17.7)
	Laborer	50 (9.2)
	Other	215 (39.)
Country of stay	United Kingdom	174 (32.1)
	Saudi Arabia	143 (26.4)
	China	117 (21.6)
	United States of America	33 (6.1)
	Canada	24 (4.4)
	United Arab Emirates	21 (3.9)
	Malaysia	17 (3.1)
	Others	13 (2.4)
Have you or any of your family members got infected with COVID-19?	Yes	93 (17.2)
	No	449 (82.8)
How are you being treated in the hospital?	Don't Know	273 (50.6)
	Free	111 (20.5)
	Good	151 (27.9)
	Not satisfied	3 (0.6)
In low-wage workers, do you think, decrease in personal income is causing anxiety and fear of unemployment?	Yes	453 (83.6)
	No	9 (1.7)
	Maybe	80 (14.8)
Are you afraid that your company will violate your contract or will end it?	Yes	165 (30.4)
	No	377 (69.6)
Are you stressed that you will not be paid due to lockdown, or will you be paid less?	Yes	267 (49.3)
	No	275 (50.7)
Do you think the lockdown is increasing your psychological stress?	Yes	469 (86.5)
	No	73 (13.5)
Are you being facilitated by the Pakistani embassy in your country of stay?	Yes	228 (42.1)
	No	314 (57.9)
Due to lockdown, have you faced visa issues (visa suspension, resolution of visa overstay and visa fee), etc.?	Yes	194 (35.8)
	No	348 (64.2)
Are you being afraid of your family members living in Pakistan being infected by coronavirus?	Yes	440 (81.2)
	No	102 (18.8)
Are you satisfied with the current management of the Pakistani government about COVID-19?	Yes	340 (62.7)
	No	202 (37.3)
Due to lack of physical activity, are you facing health issues?	Yes	221 (40.8)
	No	321 (59.2)
Do you think the people who have no company responsible for their health issues, for example, women who work as in-house maids are willing to go back to their country?	Yes	170 (31.4)
	No	132 (24.4)
	Maybe	240 (44.3)

**Figure 1:**
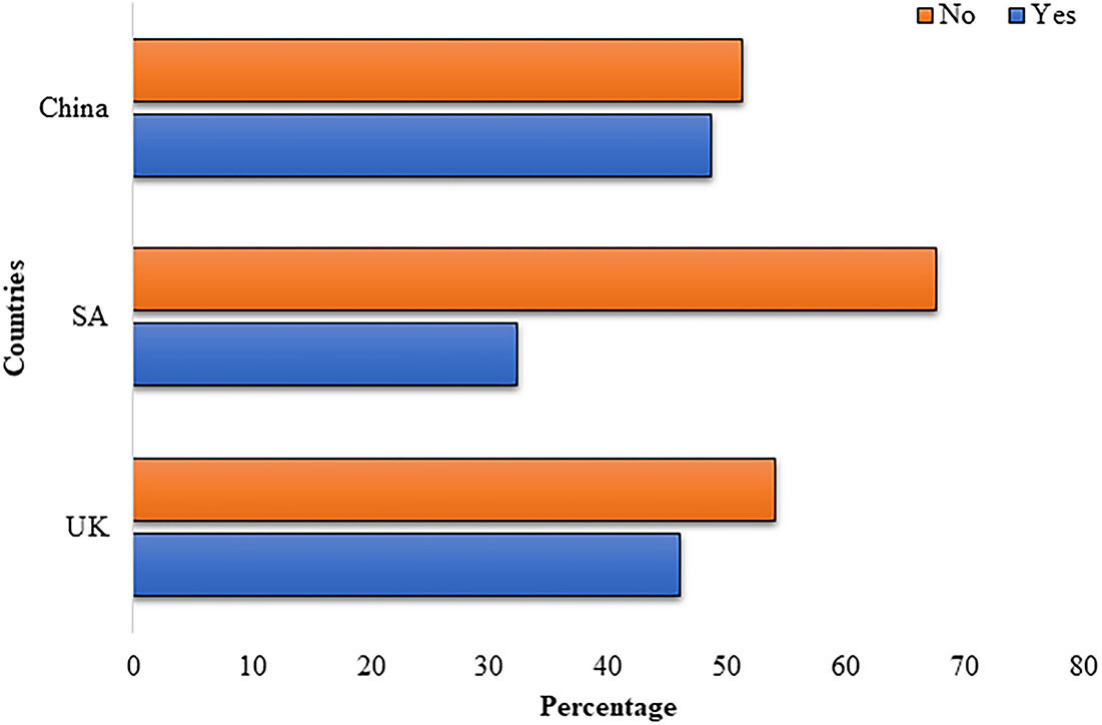
Are you being facilitated by the Pakistani embassy in your country of stay?

A small number of our study participants, or their family members 93 (17.2%), got infected with the COVID-19. Half of the study participants, 273 (50.6%), were not aware of hospital treatment quality. About one-quarter of the respondents, 151 (27.9%), stated hospital treatment is good. Most of the participants, 453 (83.6%), believed that a decrease in personal income is causing anxiety and fear of unemployment in low-wage workers. About one-third, 165 (30.4%), of the respondents were afraid that their company would violate their contracts or lose their job. Almost half of the participants, 267 (49.3%), were stressed that they are being paid less due to lockdown. The majority, 469 (86.5%), believed that the lockdown is increasing their psychological stress. More than half of the participants, 314 (57.9%), stated that the Pakistani embassy did not facilitate them in their country of stay (Figure 1).

**Figure 2:**
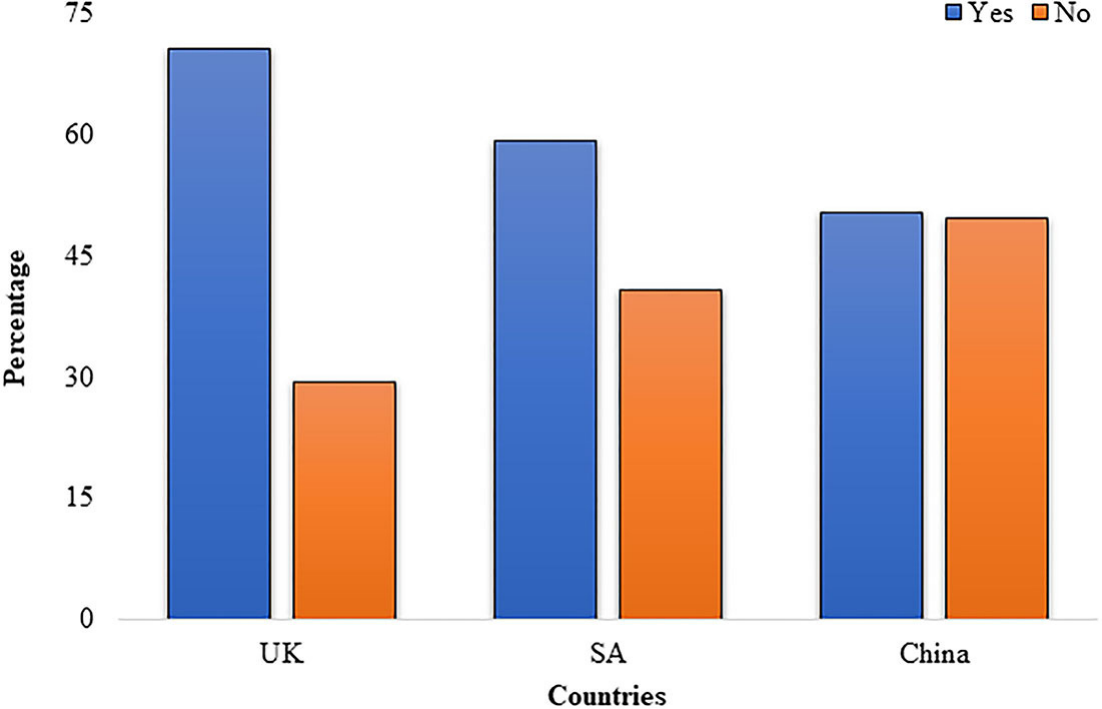
Are you satisfied with the current management of the Pakistani government about COVID-19?

About one-third, 194 (35.8%), of the respondents faced visa-related issues. Most of the participants, 440 (81.2%), worried that their family members living in Pakistan would be infected by coronavirus. About two-thirds of the participants, 340 (62.7%), were satisfied with the current management of the Pakistani government about COVID-19 (Figure 2).

More than one-third of respondents, 221 (40.8%), faced health issues due to a lack of physical activities during the lockdown. One-third of the respondents stated that the people who have no company responsible for their health insurance should return to their country (Table 1).

In total, majority of the male participants were doctors, laborers, and businessmen, while majority of the females were teachers. More males were afraid that their company would violate their contract, lose jobs, or be paid less than females (*p* < 0.001). Both genders had self-perceived psychological stress and health issues because of the pandemic. Females were more satisfied than males with the current management of the Pakistani government about COVID-19 (*p* = 0.022) (Table 2).

**Table 2:** Gender-wise comparison of education, employment status, country of stay and participants' perceptions.

**Variables**		**Females *N* (%)**	**Males *N* (%)**	***p*-value**
Education	College	13 (8.3)	40 (10.4)	0.002
	Graduate	56 (35.7)	82 (21.3)	
	Postgraduate	88 (56.1)	263 (68.3)	
Employment status	Doctor	34 (21.7)	94 (24.4)	
	Businessman	1 (0.6)	52 (13.5)	0.000
	Teacher	39 (24.8)	57 (18.8)	
	Laborer	3 (1.9)	47 (12.2)	
	Other	80 (51)	135 (35.1)	
Country of stay	UK	174 (32.1)	174 (32.1)	
	Saudi Arabia	143 (26.4)	143 (26.4)	
	China	117 (21.6)	117 (21.6)	
	USA	33 (6.1)	33 (6.1)	
	Canada	24 (4.4)	24 (4.4)	
	Malaysia	17 (3.1)	17 (3.1)	
	UAE	21 (3.9)	21 (3.9)	
	Others	13 (2.4)	13 (2.4)	
Have you or your family member got infected with COVID-19?	Yes	37 (23.6)	56 (14.5)	0.012
	No	120 (76.4)	329 (85.5)	
How are you being treated in the hospital?	Don't Know	66 (42)	207 (53.7)	0.021
	Free	48 (30.6)	67 (17.4)	
	Good	42 (26.8)	108 (28.1)	
	Not satisfied	1 (0.6)	2 (0.5)	
In low-wage workers, do you think, decrease in personal income is causing anxiety and fear of unemployment?	Yes	124 (79)	329 (85.5)	
	No	3 (1.9)	6 (1.6)	0.176
	Maybe	30 (19.1)	50 (13)	
Are you afraid that your company will violate your contract or will end it?	Yes	25 (15.9)	140 (36.4)	
	No	132 (84.1)	245 (63.6)	0.000
Are you stressed that you will not be paid due to lockdown, or will you be paid less?	Yes	58 (36.9)	209 (54.3)	
	No	99 (63.1)	176 (45.7)	0.000
Do you think the lockdown is increasing your psychological stress?	Yes	135 (86)	334 (86.8)	
	No	22 (14)	51 (13.2)	0.813
Are you being facilitated by the Pakistani embassy in your country of stay?	Yes	58 (36.9)	170 (44.2)	
	No	99 (63.1)	215 (55.8)	0.123
Due to lockdown, have you faced issues related to visa (visa suspension, resolution of visa overstay and visa fee), etc.?	Yes	59 (37.6)	135 (35.1)	
	No	98 (62.4)	250 (64.9)	0.580
Are you being afraid of your family members living in Pakistan being infected by coronavirus?	Yes	117 (74.5)	323 (83.9)	
	No	40 (25.5)	62 (16.1)	0.011
Are you satisfied with the current management of the Pakistani government about COVID-19?	Yes	106 (67.5)	234 (60.8)	0.141
	No	51 (32.5)	151 (39.2)	
Due to lack of physical activity, are you facing health issues?	Yes	63 (40.1)	158 (41)	
	No	94 (59.9)	227 (59)	0.845
Do you think the people who have no company responsible for their health issues, for example, women who work as in-house maids should go back to their country?	Yes	43 (27.4)	127 (33)	
	No	53 (33.8)	79 (20.5)	0.005
	Maybe	61 (38.9)	179 (46.5)	

The study data were analyzed country-wise by including people from three countries, i.e., UK, SA, and China, because more than a hundred participants were in each group. More females participated from UK and SA compared to China in the survey, and more males were from China compared to the UK and SA (*p* < 0.001). There were more undergraduate subjects from SA compared to UK and China. There were more postgraduates from UK and China compared to SA (*p* < 0.001) (Table 3).

**Table 3:** Country-wise comparison of participant's socio-demographic characteristics and perceptions.

**Variables**		**UK *N* (%)**	**SA *N* (%)**	**China *N* (%)**	***P*-value**
Gender	Female	65 (37.4)	57 (40.1)	1 (0.9)	0.000
	Male	109 (62.6)	85 (59.9)	116 (99.1)	
Education	College	13 (7.5)	25 (17.6)	1 (0.9)	0.000
	Graduate	52 (29.9)	41 (28.9)	19 (16.2)	
	Postgraduate	109 (62.6)	76 (53.5)	97 (82.9)	
Employment status	Doctor	41 (23.6)	34 (23.9)	24 (20.5)	
	Businessman	26 (14.9)	9 (6.3)	2 (1.7)	0.000
	Teacher	35 (20.1)	32 (22.5)	18 (15.4)	
	Laborer	19 (10.9)	17 (12)	1 (0.9)	
	Other	53 (30.5)	50 (35.2)	72 (61.5)	
Have you or any of your family members got infected with COVID-19?	Yes	40 (23)	32 (22.5)	6 (5.1)	0.000
	No	134 (77)	110 (77.5)	111 (94.9)	
How are you being treated in the hospital?	Don't Know	81 (46.6)	45 (31.7)	86 (73.5)	0.000
	Free	37 (21.3)	70 (49.3)	0 (0)	
	Good	55 (31.6)	25 (17.6)	28 (23.9)	
	Not satisfied	0 (0)	2 (1.4)	1 (0.9)	
In low-wage workers, do you think, decrease in personal income is causing anxiety and fear of unemployment?	Yes	142 (81.6)	120 (84.5)	99 (84.6)	
	No	2 (1.1)	2 (1.4)	2 (1.7)	0.904
	Maybe	30 (17.2)	20 (14.1)	16 (13.7)	
Are you afraid that your company will violate your contract or will end it?	Yes	34 (19.5)	53 (37.3)	51 (43.6)	
	No	140 (80.5)	89 (62.7)	66 (56.4)	0.000
Are you stressed that you will not be paid or paid less due to lockdown?	Yes	53 (30.5)	99 (69.7)	63 (53.8)	
	No	121 (69.5)	43 (30.3.7)	54 (46.2)	0.000
Do you think the lockdown is increasing your psychological stress?	Yes	154 (88.5)	130 (91.5)	100 (85.5)	
	No	20 (11.5)	12 (8.5)	17 (14.5)	0.306
Due to lockdown, have you faced issues related to visa (visa suspension, resolution of visa overstay and visa fee), etc.?	Yes	62 (35.6)	63 (44.4)	35 (29.9)	
	No	112 (64.4)	79 (55.6)	82 (70.1)	0.051
Are you being afraid of your family members living in Pakistan being infected by coronavirus?	Yes	130 (74.7)	125 (88)	105 (89.7)	
	No	44 (25.3)	17 (12)	12 (10.3)	0.001
Due to lack of physical activity, are you facing health issues?	Yes	57 (32.8)	67 (47.2)	58 (49.6)	
	No	117 (67.2)	75 (52.8)	59 (50.4)	0.005
Do you think the people who have no company responsible for their health issues, for example, women who work as in-house maids, should go back to their country?	Yes	80 (46)	20 (14.1)	34 (29.1)	
	No	32 (18.4)	73 (51.4)	8 (6.8)	0.000
	Maybe	62 (35.6)	49 (34.5)	75 (64.1)	

A significantly higher number of study participants suffered from COVID-19 from UK and SA compared to China (*p* < 0.001). The participants from the UK praised the quality of hospital treatment compared to KSA and China (*p* < 0.001). The participants from all three countries believed that decreasing personal income among low-wage workers is causing anxiety and fear of unemployment (*p* = 0.904).

Most of the participants from the UK were satisfied that their company would not violate their contract or terminate them, or they will be paid less compared to SA and China (*p* < 0.001). The participants from all three countries admitted that this pandemic is increasing their psychological stress (*p* = 0.306). The participants from SA were less satisfied with the efforts of the Pakistani embassy to facilitate them in their country of stay compared to the UK and China (*p* = 0.013). The participants from SA faced more issues related to visas compared to UK and China (*p* = 0.051). Compared to the UK, most of the participants from SA and China were worried that their family members living in Pakistan would be infected with COVID-19 (*p* = 0.001). Participants from the UK were more satisfied with the current management of the Pakistani government about COVID-19 (*p* = 0.002), and they were facing fewer health issues due to lack of physical activities (*p* = 0.005) (Table 3).

## Discussion

The majority of participants (83.6%) believed that decreasing personal income causes anxiety and fear of unemployment in low-wage workers. Approximately one-third of those polled were concerned that their company would breach their contracts and cause them to lose their jobs. Almost half of the participants were concerned that they were being paid less because of the lockdown. The majority of people believed that the lockdown was increasing their psychological stress. Additionally, because of the pandemic, both genders experienced psychological stress and health problems.

A study among Indian expats working in SA, UAE, Qatar, and other GCC countries reported higher anxiety and depression among the participants because of COVID 19 pandemic effects. During the pandemic, more than one-third of the participants were affected by the ban on air travel (15). They also reported a significant association between subjective concern with air traffic restrictions during the COVID-19 pandemic and the level of anxiety and depression (15). Some studies reported a higher level of depression among expats during the COVID-19 pandemic than previous studies conducted among the expat population prior to the COVID-19 pandemic (15, 16).

Our results are similar to Saudi studies that observed a high prevalence of psychological symptoms among expatriates during the COVID-19 pandemic (9, 17). This demonstrates the pandemic's significant impact on peoples' mental health, particularly among expatriates. A study from UAE, explained that the government's preventive measures and several restrictions imposed for the public were the most likely reasons for expatriate worries and stress. Since the pandemic, expats' residents have been unable to travel to their home countries on vacation. They have been unable to participate in any outdoor group activities on weekends or in their spare time (18). It seems that because of the prolong lockdown and travel ban, they were confined to their residences, which also affected their mental health.

Most overseas Pakistanis recognized and valued the measures taken by SA, UK, and Chinese governments to support and deal with the pandemic. Despite this, anxiety, stress, and uncertainty were reported. Similar findings are reported among UAE expatriates' residents (18). One-third of those polled had visa issues. It happened because all countries shut down their flight operations and imposed a travel ban on their citizens. Several people who wanted to travel to their home country for vacation or to attend some events encountered visa issues and could not travel. Most overseas Pakistani children study in Pakistan or other countries, and the travel ban has caused them to be stranded, adding to their anxiety and stress. Furthermore, their visa had expired. As a result, there were numerous factors exacerbating anxiety among overseas workers. Most overseas Pakistanis were concerned that their family members in Pakistan would become infected with coronavirus. This could be because people in Pakistan were not strictly adhering to World Health Organization (WHO) guidelines; they had inadequate knowledge and were not strictly adhering to social distancing and ignoring the severity of the issue (19, 20).

The Pakistani government took great initiatives didn't go for complete lockdown despite severe opposition political parties' pressure. The federal government resisted the call of complete lockdown in the country because of the extreme poverty and abundance of daily wages workers (5). We inquired about the Pakistani government's efforts to manage COVID-19 in Pakistan because, as overseas Pakistanis, they are in an advantageous position to compare the Pakistani government's efforts with their country of residence. Furthermore, they maintain constant contact with family members, relatives, and friends in Pakistan, and they are aware of the Pakistani government's efforts through news channels and social media. About two-thirds of the overseas Pakistanis were satisfied with the Pakistani government's efforts to manage COVID-19 in Pakistan.

More than one-third of respondents reported health problems due to lack of physical activities during the lockdown. Initially, SA, UK, and China imposed complete lockdown. People had extremely limited movement, and they were only allowed to go to nearby grocery stores or bakeries to buy their daily needs, and because of extreme fear, people were going out of home once a week, or fortnightly. This physical inactivity, combined with the extreme fear of contracting a coronavirus infection, caused widespread psychological distress. People were also afraid to go to hospitals for routine checkups or minor problems for fear of contracting a coronavirus infection (21). The daily news on social media about coronavirus infection and deaths added to their anxiety, and they were confronted with many health-related issues (22).

In comparison to China, the UK and SA had a significantly higher number of overseas Pakistanis infected with COVID-19. In comparison to SA and China, the participants from the UK praised the quality of hospital treatment. Despite the fact that the COVID-19 began in China, the Chinese government handled it meticulously after the first wave. Then, due to isolated incidents, the total death and infection rate remained low compared to other parts of the world (23).

Our study participants' response regarding the quality of hospital treatment can be explained based on a WHO report that stated that the UK healthcare system is ranked 18^th^ in the world, while SA and China are ranked 26^th^ and 144^th^ respectively (24). The UK is ranked the best healthcare system in the world overall in a 2017 report by the Commonwealth Fund ranking developed-country healthcare systems (25).

The Pakistani embassy provided the early services and facilitated (issues like visa, passport renewal, national ID card renewal, and so on) 48.7%, 32.4%, and 46% of the study participants from China, SA, and the UK repectively. In comparison to the UK and China, participants from the SA were less satisfied with the Pakistani embassy's efforts to assist them. Participants from the SA encountered more visa issues than those from the UK and China. Our results are reflected by a few months back Pakistani governments step to call back ambassador and six officers of Pakistan Embassy in SA over Pakistani community complaints (26). Many Pakistan expats working in SA and their children are studying in Pakistan or abroad, and during the COVID-19 pandemic, all have faced visa and travel problems.

Participants from all three countries agreed that a drop in personal income among low-wage workers causes anxiety and fear of losing their jobs. A study reported that expats were terrified of losing their jobs and running into financial difficulties (18). As a result of living with the coronavirus, people are understandably concerned about their health. A study reported two major reasons for this rampant and severe fear; (a) the lack of a curative drug available to date, the fear of health consequences is growing and becoming a major concern, (b) the increasing number of deaths worldwide. As a result, even those who have not been infected with the virus are concerned about their health and wellbeing, and another deeply felt concern is the threat of job loss and a financial crisis (18).

The COVID-19 pandemic imposed a prolonged lockdown period, wreaking havoc on the business and economy and displacing thousands of people (27). Therefore, the probability of job loss all over the world has been high. It has also become extremely difficult for people to find new jobs due to the coronavirus's destruction of the economy. This resulted in high levels of job insecurity for expatriates (18).

Several studies have reported that the fear of job loss and financial loss are significant sources of stress among people living in pandemic conditions in Pakistan, India, and SA (14, 28, 29). Our results are similar to studies from UAE and SA. A study from UAE reported that most Asian respondents were more severely worried about job insecurity than the Arab and Western expatriates (18). Asian expatriates may not have a good chance for new appointments, job-shifting, or re-employment in the emerging declining labor market conditions caused by the COVID-19 pandemic. As a result, the Asian community was more concerned about their job prospects than the Arab or Western expatriate residents (18). Asian expatriates in SA were also worried about the loss of jobs (29).

The pandemic's health risks are beginning to recede in some areas, and the people are being vaccinated, but still, people have hesitancy about getting the vaccine (30), and it is unclear how long the crisis will last and its long-term impact. New ways of working remotely and concerns about the global recession will continue to create uncertainty (8). Many people were worried about the health consequences, fear of losing jobs, restrictions on international travel, and fear of isolation. Even when an efficient governmental system for preventing and controlling the pandemic is in place, the difficulties that the coronavirus has presented continue to ravage people's psychological wellbeing (18).

Although the development of vaccines and widespread immunization campaigns provide some solace, the psychological scars of the pandemic are likely to last for a long time (31, 32). The most pressing and critical need in this situation is to provide financial and social security to workers abroad and those who have returned to their home countries (10). It is suggested that to alleviate stress, anxiety, and devastating effects of COVID-19 in overseas Pakistanis, they should be connected with family and friends and practice meditation and regular exercise. Yoga and meditation are also thought to be effective methods of increasing positive energy and improving mental health. Regular meditation or yoga practice may help emotionally sensitive people become calmer, more relaxed, and more stable (18). The government of Pakistan should take immediate steps to help overseas Pakistanis problems through community help and support centers in consulate offices.

## Limitations

There are several limitations to the present study. Firstly, its sample size is not large enough. Secondly, sampling techniques and online collection of data have selection bias. Thirdly, in such types of studies, no cause-and-effect relationship can be drawn. Fourthly, we did not use any psychological inventory to measure study participants stress and anxiety.

## Conclusion

Our findings show that the COVID-19 pandemic affected the overseas Pakistani's residing in the UK, SA, China, and other parts of the world. Participants in the study encountered health-related issues, and visa problems. They were dissatisfied with Pakistani embassy facilitations. Overseas Pakistanis want government aid to settle their problems.

## Data Availability Statement

The original contributions presented in the study are included in the article/supplementary material, further inquiries can be directed to the corresponding author.

## Ethics Statement

This study was approved by the Ethical Committee Abbottabad University of Science and Technology (Registration No. F.No/AUST/micro/0460).

## Author Contributions

TA, BM, and FA: conceptualization, study design, and questionnaire. TA, BM, FA, MM, MB, AI, FB, JB, MS, and AS: data collection. TA and MB: preparation of first draft, data analysis, and editing and proofreading. BM, FA, MM, AI, FB, JB, MS, and AS: helped in manuscript writing. TA: supervised the study. All authors significantly contributed to this study and approved the final manuscript for publication.

## Conflict of Interest

The authors declare that the research was conducted in the absence of any commercial or financial relationships that could be construed as a potential conflict of interest.

## Publisher's Note

All claims expressed in this article are solely those of the authors and do not necessarily represent those of their affiliated organizations, or those of the publisher, the editors and the reviewers. Any product that may be evaluated in this article, or claim that may be made by its manufacturer, is not guaranteed or endorsed by the publisher.

## Abbreviations

COVID-19: Coronavirus Disease 2019
GCC: Gulf Cooperation Council
SA: Saudi Arabia
SARS-CoV-2: Severe Acute Respiratory Syndrome Coronavirus 2
UAE: United Arab Emirates
UK: United Kingdom
USA: United States of America
WHO: World Health Organization.

## References

[1] Hu B Guo H Zhou P Shi ZL. Characteristics of SARS-CoV-2 and COVID-19. Nat Rev Microbiol. (2021) 19:141–54. 10.1038/s41579-020-00459-7 33024307 PMC7537588

[2] Khan FM Ahmad T Gulistan M Chammam W Khan M Hui J. Epidemiology of coronaviruses, genetics, vaccines, and scenario of current pandemic of coronavirus disease (2019) (COVID-19): a fuzzy set approach. Hum Vaccin Immunother. (2021) 17:1296–303. 10.1080/21645515.2020.1798697 33720797 PMC8078768

[3] Ahmad T Haroon Dhama K Sharun K Khan FM Ahmed I. Biosafety and biosecurity approaches to restrain/contain and counter SARS-CoV-2/COVID-19 pandemic: a rapid-review. Turk J Biol. (2020) 44:132–45. 10.3906/biy-2005-63 32595350 PMC7314504

[4] Poudel K Subedi P. Impact of COVID-19 pandemic on socio-economic and mental health aspects in Nepal. Int J Soc Psychiatry. (2020) 66:748–55. 10.1177/0020764020942247 32650687 PMC7443960

[5] Ali R Jawed S Baig M Azam Malik A Syed F Rehman R. General public perception of social media, impact of covid-19 pandemic, and related misconceptions. Disaster Med Public Health Prep. (2021). 10.1017/dmp.2021.229. [Epub ahead of print]. 34261567 PMC8446590

[6] Salik KM. Remittances and COVID-19: Is Pakistan ready for a likely decline in flows? © Sustainable Development Policy Institute (2020). Available online at: http://hdl.handle.net/11540/11772

[7] Year Book 2017–2018. Ministry of Overseas Pakistanis and Human Resource Development, Islamabad, Government of Pakistan (2019). Available online at: http://ophrd.gov.pk/SiteImage/Downloads/Year-Book-2017-18.pdf

[8] Mello SF Tomei PA. The impact of the COVID-19 pandemic on expatriates: a pathway to work-life harmony? Glob Bus Organ Excell. (2021) 40:6–22. 10.1002/joe.22088

[9] Algarni MA Alzahrani MS Alatawi Y Alasmari RA Alsaab HO Almalki AH. Perception of threat and psychological impact of COVID-19 among expatriates in Makkah Region, Saudi Arabia. Int J Environ Res Public Health. (2021) 18:6650. 10.3390/ijerph18126650 34205608 PMC8296444

[10] Karim MR Islam MT Talukder B. COVID-19's impacts on migrant workers from Bangladesh: in search of policy intervention. World Dev. (2020) 136:105123. 10.1016/j.worlddev.2020.105123 32834394 PMC7395589

[11] Zhang WR Wang K Yin L Zhao WF Xue Q Peng M. Mental health and psychosocial problems of medical health workers during the COVID-19 epidemic in China. Psychother Psychosom. (2020) 89:242–50. 10.1159/000507639 32272480 PMC7206349

[12] Baig M Jameel T Alzahrani SH Mirza AA Gazzaz ZJ Ahmad T. Predictors of misconceptions, knowledge, attitudes, and practices of COVID-19 pandemic among a sample of Saudi population. PLoS ONE. (2020) 15:e0243526. 10.1371/journal.pone.0243526 33296420 PMC7725365

[13] Noreen K Rubab ZE Umar M Rehman R Baig M Baig F. Knowledge, attitudes, and practices against the growing threat of COVID-19 among medical students of Pakistan. PLoS ONE. (2020) 15:e0243696. 10.1371/journal.pone.0243696 33306712 PMC7732088

[14] Varshney M Parel JT Raizada N Sarin SK. Initial psychological impact of COVID-19 and its correlates in Indian Community: an online (FEEL-COVID) survey. PLoS ONE. (2020) 15:e0233874. 10.1371/journal.pone.0233874 32470088 PMC7259495

[15] Uvais NA Nalakath MJ Shihabudheen P Hafi NAB Rasmina V Salman CA. Psychological distress during COVID-19 among Malayalam-speaking Indian expats in the middle east. Indian J Public Health. (2020) 64:S249–50. 10.4103/ijph.IJPH_475_20 32496269

[16] Sarwani SAAL Abdulla KB Mandeel MAJ. Prevalence of stress, anxiety and depression among expatriate workers. Bahrain Med Bull. (2013) 35:126–9. 10.12816/0000751

[17] Jalal SM Chacko SK David MS Khamseen ZM. Stress due to Travel Ban for Pandemic during Vacation among Expatriates of Saudi Arabia. Int J Nurs. (2021) 9:26–35. 10.37506/ijonc.v9i1.13998

[18] Baburajan P. Psychological impact of COVID-19 pandemic among expatriate residents in the UAE. Avicenna. (2021) 2021:3. 10.5339/avi.2021.3

[19] Tariq S Tariq S Baig M Saeed M. Knowledge, awareness, and practices regarding the novel coronavirus among a sample of a Pakistani population: a cross-sectional study. Disaster Med Public Health Prep. (2020). 10.1017/dmp.2020.408. [Epub ahead of print]. 33092681 PMC7943951

[20] Rehman R Jawed S Ali R Noreen K Baig M Baig J. COVID-19 pandemic awareness, attitudes, and practices among the Pakistani general public. Front Public Health. (2021) 9:588537. 10.3389/fpubh.2021.588537 34178907 PMC8219954

[21] Özdin S Bayrak Özdin S. Levels and predictors of anxiety, depression and health anxiety during COVID-19 pandemic in Turkish society: the importance of gender. Int J Soc Psychiatry. (2020) 66:504–11. 10.1177/0020764020927051 32380879 PMC7405629

[22] Gao J Zheng P Jia Y Chen H Mao Y Chen S. Mental health problems and social media exposure during COVID-19 outbreak. PLoS ONE. (2020) 15:e0231924. 10.1371/journal.pone.0231924 32298385 PMC7162477

[23] COVID-19 Dashboard by the Center for Systems Science and Engineering (CSSE) at John Hopkins University (JHU) John Hopkins University & Medicine. Available online at: https://coronavirus.jhu.edu/map.html (accessed August 17, 2021).

[24] Tandon A Murray CJ Lauer JA Evans DB. Measuring overall health system performance for 191 countries. Geneva: World Health Organization (2000). Available online at: https://www.who.int/healthinfo/paper30.pdf

[25] Mirror Mirror 2017: International Comparison Reflects Flaws Opportunities for Better U.S. Health Care. The Commonwealth Fund. Fund Reports July 14 (2017). Available online at: https://www.commonwealthfund.org/publications/fund-reports/2017/jul/mirror-mirror-2017-international-comparison-reflects-flaws-and (accessed August 20, 2021).

[26] Siddiqui N. Ambassador, 6 officers of Pakistani embassy in Riyadh called back over public complaints. Dawn, Published April 29 (2021). Available online at: https://www.dawn.com/news/1621058/ambassador-6-officers-of-pakistani-embassy-in-riyadh-called-back-over-public-complaints (accessed August 20, 2021).

[27] Ahmad T Haroon Baig M Hui J. Coronavirus disease (2019) (COVID-19) pandemic and economic impact. Pak J Med Sci. (2020) 36:S73–8. 10.12669/pjms.36.COVID19-S4.2638 32582318 PMC7306969

[28] Asim H Bokhari H Shaukat A Rehan A Rauf A Chaudhry ZA. Psychological impact of COVID-19: a systematic review. Pakistan Journal of Radiology. (2020) 30:282–5.

[29] Al Sulais E Mosli M AlAmeel T. The psychological impact of COVID-19 pandemic on physicians in Saudi Arabia: a cross-sectional study. Saudi J Gastroenterol. (2020) 26:249–55. 10.4103/sjg.SJG_174_20 32496223 PMC7739997

[30] Alzahrani SH Baig M Alrabia MW Algethami MR Alhamdan MM Alhakamy NA. Attitudes toward the SARS-CoV-2 vaccine: results from the Saudi residents' intention to get vaccinated against COVID-19 (SRIGVAC) study. Vaccines. (2021) 9:798. 10.3390/vaccines9070798 34358214 PMC8310025

[31] Ahmad T Murad MA Baig M Hui J. Research trends in COVID-19 vaccine: a bibliometric analysis. Hum Vaccin Immunother. (2021) 17:2367–72. 10.1080/21645515.2021.1886806 33687303 PMC8475596

[32] Serafini G Parmigiani B Amerio A Aguglia A Sher L Amore M. The psychological impact of COVID-19 on the mental health in the general population. QJM. (2020) 113:531–7. 10.1093/qjmed/hcaa201 32569360 PMC7337855

